# Parenting Stress and Social Style in Mothers and Fathers of Children with Autism Spectrum Disorder: A Cross-Cultural Investigation in Italy and Japan

**DOI:** 10.3390/brainsci11111419

**Published:** 2021-10-27

**Authors:** Michele Giannotti, Sophia Marlene Bonatti, Sanae Tanaka, Haruyuki Kojima, Simona de Falco

**Affiliations:** 1 Laboratory of Observation, Diagnosis and Education (ODFLab), Department of Psychology and Cognitive Science, University of Trento, 38122 Trento, Italy; sophia.bonatti@unitn.it (S.M.B.); simona.defalco@unitn.it (S.d.F.); 2 Research Center for Child Mental Development, Kanazawa University, Kanazawa 920-0925, Japan; tanakast@staff.kanazawa-u.ac.jp (S.T.); hkojima@staff.kanazawa-u.ac.jp (H.K.); 3 Faculty of Human Sciences, Kanazawa University, Kanazawa 920-1192, Japan

**Keywords:** autism spectrum disorder, parenting stress, parenting style, cross-cultural, fathers

## Abstract

Parents of children with autism spectrum disorder (ASD) face unique challenges, which may affect parenting functioning. However, little is known about gender and cultural variations in parenting stress and styles in these families. The aims of this study were to investigate: (1a) the differences in parenting stress and (1b) social style between Italian and Japanese mothers and fathers of children with ASD; (2) the predictive role of culture, sociodemographic, and child’s characteristics on parenting stress; (3) the predictors of the social parenting style, including parenting stress dimensions. The study involved 92 Italians and 89 Japanese parents of school-age children (5–12 years) with ASD who completed the Parenting Stress Index and the Parenting Style Questionnaire. Results revealed that Japanese parents showed higher parenting stress and less engagement in social style than Italians. Across cultures, mothers used more social style than fathers. Being Japanese and having a child with greater ASD severity predicted higher levels of parenting stress. We also found that country, parent’s gender, and stress related to the dysfunctional interaction were significant predictors of parenting social style. Our findings highlight the importance of a cross-cultural approach to better understand the experiences and needs of mothers and fathers of children with ASD.

## 1. Introduction

Autism spectrum disorder (ASD) is a complex life-long and early-onset neurodevelopmental disorder characterized by persistent impairments in social communication and interaction across multiple contexts, as well as restricted and repetitive patterns in behaviors, interests, and activities [1]. In light of these characteristics, both mothers and fathers of children with ASD could face unique challenges and parenting demands, bearing consequences also on their psychological distress [2,3]. In particular, literature has documented that parent–child dyadic exchanges in ASD can be characterized by recurrent mismatches, less sustained positive emotions, and non-sequential patterns of interaction [4,5]. These relational impasses could make it more difficult for the parents to interpret and understand the child’s intentions and affective needs, constituting a considerable source of stress. Therefore, parents may often perceive the interaction with their child as unrewarding and challenging, experiencing negative feelings, which can significantly increase parental stress and mental health difficulties [6,7]. Higher parental stress and mental health problems in parents of children with ASD can pose further risks for parent–child relationships generating a cascade of adverse effects on parenting functioning [8,9].

### 1.1. Parenting a Child with Autism Spectrum Disorder in Italy and Japan: A Cross-Cultural Perspective

Parenting refers to the process of promoting the social, physical, emotional, and intellectual development of a child [10], providing a supportive and nurturing early social environment through adequate and prompt sensitive behaviors [11].

According to a processual model [12], parenting is determined by the complex interplay between several factors, such as children’s and parents’ characteristics and social-contextual dimensions. Focusing on the social–contextual level, the “socialization process” carried out by parents is deeply influenced by the culture in which they are immersed [13]. Based on Hofstede’s cultural dimensions theory [14], Japanese culture is considered a collectivistic society, since the harmony and the interest of the group tend to prevail over the individuals’ ones. In such societies, social harmony is highly valued, and individuals are expected to inhibit the expression of their own needs and attend to the needs of others in their in-group [15]. Parents in collectivistic cultures try to promote these values through parenting practices that encourage children to view themselves as part of the integrated whole of their in-group (family, community, and society), and not to emphasize their differences from others [16,17,18,19]. On the other hand, Italian culture is defined as individualistic, since the interests of the individual overcome the benefits of the group. In individualist societies, independence, self-interest, and self-reliance are highly valued in the socialization process. Accordingly, parents tend to promote autonomy, exploration, and uniqueness in their children, and the individual’s sense of identity is expected to develop more according to personal characteristics than in conformity to their group membership [14,15].

These cultural differences are also reflected in the way societies and families deal with children with atypical development. In the individualist communities, the dominant philosophy is to treat these children as much as possible as children with typical development (TD), whereas in the collectivist communities the disability is more susceptible to stigmatization and could be seen as a shame for the family [14,15], as children’s behaviors and attitudes are considered as a direct reflection of the parents’ child-rearing capabilities.

These distinct cultural views also contribute to the differences in health systems and social inclusion strategies for children with special needs. For instance, while in Italy, most children with special needs, including children with intellectual disabilities, are integrated into the regular school system, in Japan, children with intellectual disabilities are encouraged to attend special schools, whereas children with special needs without intellectual disabilities join regular schools where they have the option to attend support classes.

Taken together, the challenges associated with rearing a child with ASD could differ among the Japanese and Italian cultures as divergent social models and policies regulate the experience of parenting children with disabilities. Even if there is a global awareness of ASD and a similar prevalence among countries, we have insufficient knowledge about its impact on children and their families in different world areas and cultures [20]. Therefore, cross-cultural comparisons may be particularly relevant, since cultural differences could not only influence the perception of child symptoms [21] but could also impact parental attitudes and behaviors, affecting several parent–child domains, including parental stress and styles.

### 1.2. Parenting Stress in Mothers and Fathers of Children with ASD

Among several parenting dimensions, most of the research concerning parenting and ASD has focused on parental stress, mainly on mothers, since they are often the child’s primary caregiver. Parenting stress is defined as a set of processes that lead to adverse psycho-physiological reactions arising from attempts to adapt to the demands of parenthood [22]. In general, literature widely showed that mothers of children with ASD experience higher levels of parenting stress than mothers of children with other neurodevelopmental disorders (e.g., Down syndrome) [23] and typical development (TD) [24]. Consistently, higher levels of parenting stress have been found both in Japanese mothers [20] and Italian [25] parents of children with ASD when compared to families of TD children.

While parenting stress findings have been well corroborated for mothers, only a few studies specifically focused also on fathers of children with ASD [26], mainly reporting higher stress levels than fathers of TD children [27] both in Italy [25,28] and Japan [29].

Of note, only a limited number of studies focused on gender differences in parenting stress, providing mixed evidence. Some findings revealed that mothers experience higher levels of stress and involvement than fathers [27,29,30,31], while others reported similar stress levels for both parents [32,33], including a study on an Italian sample [28].

### 1.3. Social Style in Mothers and Fathers of Children with ASD

Parenting styles are defined as the constellation of attitudes, expectations, and beliefs concerning the upbringing of a child that results in a set of parental behaviors used in the daily interactions with their own offspring [34]. Specifically, social style refers to parenting behaviors in the context of parent–child dyadic interpersonal exchanges, involving the expression of parental sensitivity, affect, and mutual reciprocity [35,36].

With respect to the Italian context, research on parenting styles indicated that mothers highly regard their children’s socioemotional expressiveness and encourage their development through the promotion of infants’ interactions with people inside and outside of the family [37]. Accordingly, Italian parents tend to value sociability, liveness, and activity in their children and declare interacting with their babies more in the social and interpersonal domain [38].

In Japan, observational studies showed that mothers engage in more social interaction [39] and less didactic activities [40] with their children than European/American mothers. Japanese mothers considerably value social competence in their infants and tendentially engage in more symbolic play and “empathy training” with their children [40,41,42].

Although the evidence that parental styles and behaviors play a key role in the development of children with ASD—as it does with TD children—is increasing, this area of research is still under-investigated [8], with the few studies comparing parental styles of parents of TD and ASD children yielding contrasting results both in Eastern and Western cultures [8,43,44,45,46,47,48,49].

Concerning gender differences in parenting styles, it has been reported that maternal and paternal behaviors diverge in various ways [11]. For instance, father involvement in infancy and childhood is quantitatively less than mother involvement [50,51]. As is the case of TD, also in families of children with ASD, mothers dispense more time with their children and have more caregiving and managerial roles compared to fathers whose interactions are characterized by play activities [28]. This also reflects differences in the strategies used by fathers and mothers of children with ASD. In general, fathers tend to be more directive and less active in engaging their children with ASD [52,53], while mothers of children with ASD reported taking on more social behaviors than fathers [28].

In addition to child diagnosis and parent gender, research suggests that parenting behaviors and styles may be regulated by parents’ cognitions and emotional states. In particular, higher levels of parenting stress have been associated with less positive behaviors in the context of parent–child dyadic interpersonal exchanges [54], particularly in parents of children with ASD [22]. A recent Chinese study conducted on families of children with ASD confirmed the significant effect of parenting stress on parenting behaviors emerged not only in mothers but also in fathers [55].

To summarize, mothers and fathers of children with ASD tend to report higher parenting stress compared to parents of children with TD, regardless of culture. Literature on gender differences related to parenting stress provides mixed findings. With respect to parenting behaviors, studies suggested that mothers engage more frequently than fathers in sensitive behaviors in the interaction with their children. Research on Eastern countries has shown that parents of children with ASD exhibit less positive parenting compared to parents of non-ASD children. However, it is still unclear whether this result is culture-specific or if Western parents of children with ASD show similar levels of social style than their Eastern counterparts.

Moreover, it is important to consider that parenting stress seems to have a significant impact on maternal and paternal behaviors, limiting the ability to provide optimal and sensitive parenting, especially in parents of children with ASD. Although literature has widely investigated maternal dimensions, particularly parenting stress, little attention has been paid to fathers of children with ASD. Other crucial parenting domains, such as parenting style, have not been sufficiently addressed so far. Importantly, both parenting stress and styles can be influenced by cultural models, since individualistic and collectivistic countries may endorse different socio-contextual views related to atypical neurodevelopment, childrearing practices, and gender roles in parenting. However, no previous studies on the families of children with ASD examined similarities and differences in parenting stress and social parenting style comparing mothers and fathers of Eastern and Western countries.

### 1.4. The Current Study

In the present study, we aimed: (1a) to investigate potential differences in parenting stress and parenting social style (1b) between Italian and Japanese mothers and fathers of children with ASD; (2) to examine the predictive role of sociodemographic characteristics (i.e., culture, parental gender, age, and family socioeconomic status), child’s features (i.e., child IQ and ASD severity) on parenting stress in parents of children with ASD in Italy and Japan; (3) to study the predictive role of culture, parents’ sociodemographic characteristics, child’s characteristics, and parenting stress on the social parenting style in Italian and Japanese parents of children with ASD.

Regarding our first aim, we expect to find higher levels of parenting stress in Japanese parents than Italian parents, due to the higher social pressure aimed at maintaining group harmony, which characterizes the collectivistic background. No gender differences were expected in both samples on parenting stress. Secondly, we hypothesized that Japanese mothers and fathers would engage less in the social domain of parenting than Italian parents, since their need to act in conformity to their group membership may hamper their engagement in the challenging parent–child interactions, encouraging the suppression of disagreeable emotional states. With respect to gender differences, we hypothesize to find higher levels of social parenting style in mothers than fathers, regardless of culture.

Concerning our second aim, we expected that country of origin and child’s characteristics would predict parenting stress of parents of children with ASD. Finally, our expectation about the third aim was that parents’ gender and parenting stress would be predictive of social parenting style in both cultures.

## 2. Materials and Method

### 2.1. Participants and Procedures

The present study involved 181 parents of school-age children (5–12 years) with a diagnosis of ASD confirmed through clinical judgment as well as through the Autism Diagnostic Observation Schedule [56,57,58]. Participants from Italy were 92 parents (47 mothers: M age = 42.12 years, SD = 5.75; 45 fathers: M age =45.19 years, SD = 5.63) of 47 children with ASD (M age = 110.94 months, SD = 23.41). They were recruited from the Observational, Diagnosis, and Training Lab (ODFLab), Department of Psychology and Cognitive Science of the University of Trento, where they had been followed for psychodiagnostic assessment and clinical interventions for neurodevelopmental disorders. The children’s IQ was in the normative range (M = 87.322, SD = 19.67) and was assessed using the Wechsler Intelligence Scales for Children (WISC-III; WISC-IV; WPPSI; [59,60,61]) or, for the verbally impaired subjects, through the Leiter International Performance Scale—Revised (Leiter-R; [62]). Family Socioeconomic level was in the medium range as measured with the Hollingshead Four-Factor Index [63]. Japanese participants were 89 parents (47 mothers: M age = 41.36, SD = 4.09; 42 fathers: M age = 44.69, SD = 6.47) of 47 children with ASD (M age = 107.42 months, SD = 25.57). They were recruited from the Research Centre for Child Mental Development of Kanazawa University where they were participating in another study (BambiPlan study). The intelligence quotient (IQ) of the Japanese children was measured through the Kaufman Assessment Battery for Children [64] and fell within the normative range (KABC; M = 93.55, SD = 19.46). Family socioeconomic level was in the medium range according to the Hollingshead Four-Factor Index [63]. All measures based on questionnaires (see Section 2.2) were collected via mail from the Japanese parents. More detailed participants’ characteristics are presented in Table 1.

Both Japanese and Italian participants provided their formal informed consent in participating in the study. Data were collected according to European current regulation (European General Data Protection Regulation—GDPR UE 2016/67) and following the Helsinki Declaration principles. As regards the Italian participants, all procedures of our study were in accordance with the ethical standards of the Italian Association of Psychology and the Ethics Committee of the Faculty of Psychology and Cognitive Science of the University of Trento (Italy). With respect to the Japanese sample, the study has been approved by the local IRB local committee (N: 2016-430 (759)).

### 2.2. Measures

#### 2.2.1. Children’s Intelligence Quotient (IQ)

For assessing children’s IQ, according to the age of the child, the Wechsler Intelligence Scale for Children (WISC-III; WISC-IV; [59,60]), Wechsler Preschool and Primary Scale of Intelligence (WPPSI; [61]), and Leiter International Performance Scale—Revised (Leiter-R [62]), alternatively to WISC, in verbally impaired subjects have been used for the Italian sample. For Japanese children, the IQ assessment was carried out with the Japanese version of the Kaufman Assessment Battery for Children [64], as this battery is more used in Japan as an IQ measure. All these scales use standard scores that have a mean of 100 and a standard deviation of 15, allowing direct IQ comparisons of intelligence. The WISC, Leiter-R, and the K-ABC results have been standardized in order to have comparable scores across instruments.

#### 2.2.2. Autism Symptoms Severity

To classify the ASD severity of the children, we used the Autism Diagnostic Observation Schedule (ADOS), and its Second Edition (ADOS—II) module’s scores. ADOS tests are semi-structured, standardized assessments of social interaction, communication, play, and imaginative use of materials for people suspected of having ASD [56,57,58]. We transformed raw scores following the manual’s protocol and calculated the diagnostic algorithm. In particular, the total score of the ADOS was confronted with the cut-offs of each ADOS module, being Module 1—from no word to very few words, are autism = 16, autism spectrum = 11; Module 1—some words, are autism = 12, autism spectrum = 8; Module 2—equal or more than 5 years, are autism = 9, autism spectrum = 8; Module 3, are autism = 9, autism spectrum = 7. From this, we generated a dichotomous variable: one including all participants scoring above the modules’ cut-offs for autism and one including all participants that were above the modules’ cut-offs for the autism spectrum but below the autism ones.

#### 2.2.3. Parenting Stress

The Parenting Stress Index—Short Form (PSI-SF; [65]) is a widely used self-report questionnaire for the assessment of parenting stress. It has been developed on account of various exploratory factor analyses of the full PSI [66], and it consists of three subscales of 12 items: parental distress (PD), parent–child dysfunctional interaction (PCDI), and difficult child (DC). The sum of the three subscales yields to a total stress (TS) scale. Specifically, the PD subscale indicates the distress felt by a parent because of personal factors related to parenting, such as low social support or parental depression (item example: “I feel alone and without friends”). The PCDI subscale evaluates if the parent sees his/her interactions with the child as positive/rewarding or negative/unsatisfying (item example: “My child smiles at me less than I expected”). The DC subscale assesses the child’s behavioral characteristics that make him/her easy or difficult to manage as a result of his/her temperament and/or, non-compliant, defiant, or demanding behavior [67] (item example “My child is moody and easily upset”). Additionally, the PSI-SF contains a defensive responding scale (DF) signaling the degree to which the parent might be trying to give a more positive self-image, deny stress, or minimize problems in their relationship with the child. Parents responded to the 36 items on a 5-point Likert scale indicating the degree of agreement with each statement, ranging from 1 (strongly agree) to 5 (strongly disagree). The TS score is a measure of the parent’s overall parenting stress. All the scales (PD, PCDI, DC, TS) have been demonstrated to have a high internal consistency [65,68]. Since the PSI-SF was not available for purchase in Japan at the time of this study, we used the Japanese PSI full version for the Japanese parents. The PSI-SF is a direct derivation of the PSI full version; for this reason, we were able to construct the PSI-SF in Japanese selecting the corresponding items from the Japanese version of the PSI-full.

#### 2.2.4. Parenting Social Style

The parental social style was assessed by the social exchange scale of the Parental Style Questionnaire (PSQ; [35,69]), a self-report questionnaire to measure individual differences in parenting behavior’s domains. For Italian parents, we used the Italian version of the PSQ [36], and for Japanese parents, an ad hoc translation was created starting from the English original version, following standard back-translation techniques and seeking for semantic, linguistic, and cultural equivalence [70]. Parents were asked to rate each item on a 5-point Likert scale ranging from 1 to 5 based on how often they display the described behavior. The social exchange scale is formed of 5 items that assess how often parents engage in dyadic interactions with their children characterized by sensitivity, expressions of affection, and positive responsiveness to the child. The scale’s score is formed by the unweighted mean of responses to the five items. An example of a question regarding the social exchange style is: “I provide my child with positive affectionate displays of warmth and attention”. The scale has shown good internal consistency and construct validity [28,35,36,38,69].

### 2.3. Data Analysis

Descriptive statistics by country are displayed in Table 2. Firstly, we checked for normality of the distribution, skewness, and kurtosis of the study variables and outliers. Then, bivariate correlations were separately performed to test the association among the study variables. Next, as preliminary analyses, we checked for potential differences between countries on categorical and continuous control variables. Specifically, we examined potential group differences between Italian and Japanese samples in child gender, autism severity (using the chi-square tests), as well as in maternal and paternal age, child age, intelligence quotient, and family socioeconomic status (using the multivariate analysis of variance).

With regards to the first aim of the study, we performed a two-way (Country × Gender) multivariate analysis of variance (MANOVA) with PSI-SF total stress and subscales (PD, PCDI, DC, TS) as dependent variables. Similarly, a two-way ANOVA (Country × Gender) has been conducted using the parenting social style scale as a dependent variable. With respect to our second aim, to investigate potential predictors of parenting stress, we performed a hierarchical multiple regression, including three blocks. In the first block, we included culture, then, parent’s gender, age, and family SES were added in the second block; next, the child’s IQ and autism severity were entered in the last block. Finally, the same regression model was used to examine potential predictors of the social parenting style, but a fourth block was added to the overall model, including the three PSI-SF subscales (PD, PCDI, DC). In general, the variables’ entrance was forced according to our research purposes. We also checked the Variance inflation factors (VIFs) for both regression models in order to determine multicollinearity among the predictors (Fox and Weisberg, 2011). Regressors with a VIF value smaller than 4 (Zuur, Ieno, and Elphick, 2010) were considered acceptable and, thus, included in the models. All the statistical analyses were conducted using the SPSS package (22.0 for Windows).

## 3. Results

### 3.1. Cross-Cultural Comparisons

Bivariate correlations among the study variables for both countries are shown in the Supplementary Materials. With respect to the group comparison on the control variables, chi-squared tests and the MANOVA yield non-significant results (*p* > 0.05), since no differences emerged respectively for child gender, autism severity, child IQ, family SES, and child, maternal, and paternal age. Focusing on the first study aim, results of the two way MANOVA (Country × Gender) revealed statistically significant differences in parenting stress based on country (F(3, 173) = 7.70, *p* < 0.001; Wilk’s Λ = 0.882, partial η^2^ = 0.12; whereas, no significant effect of parent gender and no interaction between country and gender were found. Specifically, univariate analysis showed a main effect of country on parental distress (F(3, 178) = 22.60, *p* < 0.001) and difficult child subscales (F(3, 178) = 6.99, *p* < 0.01) as well as on total stress scale, (F(3, 178) = 14.16, *p* < 0.001), with Japanese parents showing higher scores compared to Italian parents. As regard to group differences in parental social style, the two-way ANOVA (Country × Gender) highlighted a main effect of the country (F(3, 180) = 8.66, *p* < 0.01), with Italian parents reporting higher scores compared to Japanese parents. Moreover, a main effect of parent gender (F(3, 180) = 12.04, *p* < 0.001) emerged, with mothers showing higher levels of parental social style than fathers.

### 3.2. Predictors of Parental Stress

According to our second aim, we performed hierarchical regression analyses to investigate the contribution of specifically selected sociodemographic, parenting, and child variables on parental stress (Table 3). The overall model explains around 13% of the variance (F(6, 177) = 4.30, *p* < 0.001). In the first step, country of origin (β = 0.265, *p* < 0.001) significantly predicted parental stress, explaining 7% of the variance, whereas the second block, including parent gender, parent age, and family SES did not add a significant contribution to the model. In the final model, there was a significant predictive effect of country (β = 0.268, *p* < 0.001) and child autism severity (β = −0.213, *p* = 0.004), adding significant contribution to the overall model (R^2^change = 0.049, *p* = 0.009). Specifically, being a Japanese parent and having a child with higher autism severity predicts higher levels of parenting stress.

### 3.3. Predictors of Parental Social Style

We replicated the above-described regression model to examine the predictors of parental social style, by adding a fourth block, including the three PSI-SF subscales (Table 4). This hierarchical regression analysis revealed that the predictors explain around 32% of the total variance (F(9, 176) = 10.27, *p* < 0.001). Country of origin was significant in the first block, with being an Italian parent associated with a higher level of parental social style (β = −0.200, *p* = 0.008). In the second block, only country of origin and parent gender were significant, whereas no predictive effect of parent age and family SES was found (F(4, 176) = 4.87, *p* = 0.001). In particular, being a mother (β = −0.253, *p* = 0.001) predicted a higher level of parental social style, adding a significant contribution to the model (R^2^change = 0.062, *p* = 0.009). Although the model remains significant (F(6, 176) = 3.44, *p* = 0.003), the third block did not add a significant contribution, since the entered variables (child IQ and autism severity) were both not significant (*p* > 0.05).

In the final model (fourth block), country of origin (β = −0.193, *p* = 0.004), parent gender (β = −0.087, *p* = 0.046), and stress related to parent–child dysfunctional interaction (β = −0.578, *p* < 0.001) showed a significant predictive effect on parental social style. Conversely, child age, parent age, family SES, child IQ, and autism severity as well as parental distress and difficult child PSI subscales did not contribute significantly to the overall model.

## 4. Discussion

Parenting stress and styles are considered two critical domains in the field of parenting and ASD, since social communication impairments may pose specific challenges for mothers and fathers, straining parent–child interaction. However, only a limited number of studies examined potential gender differences in parenting stress and style in families of children with ASD, providing contradictory results. Moreover, although these two dimensions can be influenced by cultural tendencies toward childrearing and atypical development, previous studies did not address potential differences between individualistic and collectivist countries focusing on families of children with ASD. Therefore, to fill this gap, this cross-cultural study aimed at investigating potential gender and cultural differences in parenting stress and social style between Italian and Japanese mothers and fathers of children with ASD. In addition, we examined the contribution of culture, child, and parenting variables that could influence parenting stress and style in the context of ASD.

### 4.1. Gender and Cross-Cultural Differences in Parenting Stress and Social Style

To the best of our knowledge, this is the first study testing potential cross-cultural differences in parenting stress and social style between Japanese and Italian parents of children with ASD.

Firstly, as hypothesized, parenting stress was found to be higher in Japanese parents compared to Italian parents, particularly on the parental distress subscale, which refers to the extent to which parents feel competent, restricted, conflicted, supported, and/or depressed in their role as a parent. This result may be ascribed to the cultural backgrounds that characterized collectivistic society, as is the case of Japan. In fact, collectivistic cultural tendencies such as trying to adjust them to a typical standard may increase their parenting stress in mothers and fathers of children with ASD. Parenting a child with atypical neurodevelopment entails various challenges that may be at odds with a collectivistic approach, which relies on attending family and community harmony [16,17,18]. In this socio-cultural context, ASD socio-communicative impairment can make adherence to group common values and norms harder to accomplish, constituting a considerable source of stress for parents. For instance, when mothers perceive themselves as ineffective in managing their children’s difficult behaviors, as may occur in the context of ASD, they are more likely to experience feelings of guilt and shame, perceiving to fail in their role in the eyes of the community and, thus, arousing parenting stress [19].

Second, no differences in parenting stress were found among mothers and fathers of children with ASD, regardless of culture. Although prior research provided mixed results, our finding is in line with other studies reporting similar levels of parenting stress in both parents [32,33], including a study conducted on an Italian sample [28]. This result confirmed the importance of including fathers in ASD early intervention [26], since they may experience higher levels of parenting stress just like mothers.

Focusing on parenting social style, our findings revealed significant cross-cultural and gender differences. According to our hypothesis, Italian parents scored higher than Japanese parents on the parenting social style scale, reporting to engage more frequently in sensitive, responsive, affectionate dyadic interactions with their children. A possible explanation of this finding is that Japanese parents could show less positive engagement with their children when they show difficult behaviors in social situations, since group membership and child social competence are prioritized instead of the child’s affectional needs. In the context of ASD, externalizing behaviors, arousal dysregulation, or exaggerated expression of negative affect may be considered unsuited for the maintenance of group harmony. Additionally, at this range of child age, Japanese parents are more likely to fulfill child independence and to express less emotional regulation than Italians.

Moreover, mothers reported higher levels of parental social style compared to fathers, regardless of culture. Our finding is in line with a previous study by Ozturk and colleagues [28] conducted on Italian mothers and fathers of children with ASD. This result may also be explained by the divergent style of parenting used by fathers, which tends to be more directive, energetic, and competitive with their children than mothers [50,51,52,71]. Furthermore, mothers are more involved in childcare than fathers, spending more time with their children [51] and, thus, having more opportunities to engage in social dyadic exchanges.

### 4.2. Predictors of Parenting Stress and Social Style in Japanese and Italian Parents of Children with ASD

With respect to parenting stress in mothers and fathers of children with ASD, country (being Japanese) and the severity of ASD symptoms emerged as the only significant predictors. First, as observed on group differences, the role of social pressure related to the socio-cultural collectivistic view may have increased the levels of perceived parenting stress in Japanese parents.

Therefore, it becomes essential to note that cultural background could have an impact on the experience of mothers and fathers, influencing the perception of their parental role and the burden associated with the caregiving practice. Moreover, Japanese parents can be more susceptible to self-blame, since their cultural background endorses a role-oriented approach in which child behaviors are considered a reflection of parental abilities and competence [72].

According to the traditional literature on parenting and ASD [32], we also found that parenting stress levels are associated with child symptoms’ severity (social and communicative impairment as well as repetitive and stereotyped behaviors and interests). In this regard, it is plausible that children with higher ASD severity show higher levels of dependency toward their parents, requiring intense caregiving support, which may escalate parental strain and worries. Thus, caring for a child with severe ASD symptoms could constitute a specific risk factor for both mothers and fathers across countries, making it more difficult to balance their psychological and coping resources with the parenting-related demands. In fact, higher impairment in interpersonal relatedness and social communication could pose additional challenges for parents, affecting their sense of competence and efficacy [73]. Additionally, previous studies [20,25] also demonstrated a significant link between parental stress and behavioral and emotional problems related to the ASD diagnosis. It is important to consider that parenting distress in Italian and Japanese parents of children with ASD has been found to be associated with parental depression, the balance of parents’ diverse roles in their life, lack of social support, attachment difficulty, anxiety about the child’s future and caregiving burden, and quality of parent–child interaction [20,28].

Furthermore, we found that country, gender, and parenting stress related to parent–child dysfunctional interaction have a predictive effect on parenting social style.

As previously highlighted for group comparison on parenting social style, the effect of the country could be ascribable to the divergent cultural approach on child-rearing, which characterize individualistic and collectivistic societies. For instance, parents coming from individualistic countries, as is the case of Italy, are focused on promoting child uniqueness, since individual development is expected to be shaped more by personal characteristics than group expectations and needs [15]. Accordingly, Italian parents reported interacting with their children more on the social and interpersonal level compared to other parenting domains [38]. Conversely, Japanese mothers and fathers could show less engagement in the social domain of parenting than Italian parents, given that the more frequent display of negative emotions associated with ASD may be in contrast with their in-group values and norms and, thus, less tolerable for parents.

Regardless of the country, we also found that mothers showed a higher parenting social style with their children than fathers. According to our hypothesis, it is likely that mothers across cultures assume more caregiving responsibilities, providing higher support to their children in the social exchange. In line with previous literature on typical and atypical development, mothers tend to engage in highly responsive behaviors toward their children in the context of dyadic interaction, promoting their socio-emotional expressiveness and competence [28,37,72]. Importantly, we also found a significant link between parenting stress related to parent–child dysfunctional interaction and parenting social style across countries. Specifically, higher maternal and paternal stress levels related to feelings of disappointment, rejection, or alienation by/from the child predict fewer expressions of affection and positive responsiveness to the child. Our findings extended previous studies conducted only on Eastern countries revealing the effect of parenting stress on maternal and paternal care in families of children with ASD [55]. Interestingly, parenting functioning in the dyadic exchanges with the child seems more related to relational stress instead of parenting stress generated by personal adjustment to parenthood or child individual difficulties. It is important to note that parenting stress in ASD is associated with more authoritarian and permissive parenting and higher levels of overprotection in ASD [46,55], which have been found to be associated with negative child outcomes in ASD [74].

Finally, no effect of child IQ and family SES on both parenting stress and social style were found. Although prior research revealed a significant effect of child IQ [75,76], other studies have shown that cognitive functioning is not a salient feature in predicting parenting stress [32]. Moreover, in our sample, most of the children showed high cognitive functioning, since they scored above 70 on the developmental assessment. Similarly, the lack of significant effect of family SES on parenting stress may be due to the poor variability of our samples on this dimension. Given that no effect of ASD symptoms severity has been observed on parenting social style, it is plausible that other variables such as child externalizing behaviors or emotion regulation processes may intervene in shaping maternal and paternal sensitive and affectional behaviors in the context of dyadic interaction.

## 5. Conclusions

The current study supports the traditional models of determinants of parenting [12,77], according to which maternal and paternal parenting are determined by multiple factors, including parent and child variables as well as socio-cultural dimensions. Firstly, our results highlight the need to evaluate the influence of cultural tendencies on parenting stress perception and dyadic interactive style also in families of children with ASD. This is of particular relevance, since the sociocultural view on child-rearing and atypical neurodevelopment may play a pivotal role in shaping parental cognitions and practice. To sum it up, our findings suggest that a collectivist cultural approach could make Japanese parents more susceptible to parental stress related to feelings of competence and restriction in their role as a parent. By contrast, Italian parents of children with ASD reported engaging more frequently in responsive and affectional behaviors compared to Japanese parents. In this case, individualistic cultural models seem to promote more affectionate parenting focused on sharing positive affect and supporting child initiative. On the other hand, parents who endorse a collectivistic approach could be more prone to minimize the expression of disagreeable effects or inadequate child initiatives to fulfill group needs and be perceived as competent by the community. Other findings are independent of culture. For instance, autism severity was found to be predictive of parenting stress for both Japanese and Italian parents.

Focusing on parenting social style, mothers reported higher levels of social exchange with their children. Parenting stress related to parent–child dysfunctional interaction strongly predicted sensitive, responsive, and affectionate parenting across countries in both mothers and fathers. The current study also has clinical implications, highlighting the importance of supporting both mothers and fathers of children with ASD through specific interventions that take into account parental cultural background. In particular, Japanese parents of children with ASD may benefit from interventions aimed at reducing self-blame and worries associated with feelings of competence and parental role strain. Fathers should also be included in supporting programs for parents of children with ASD, since they showed similar levels of parenting stress to mothers. Tailored parent-mediated interventions are recommended to boost parent–child interaction in ASD and to decrease feelings of rejection and alienation in parents. This could be of relevance in helping parents to handle child dysfunctional behaviors in the context of dyadic interaction.

Notably, our findings should be interpreted with caution, since some limitations need to be acknowledged. The heterogeneity of our sample in terms of child age, cognitive functioning, and ASD severity may constitute a limitation. In addition, we dichotomized the ASD severity variable due to the multiple versions and modules used for the assessment, and thus, it was not possible to use a continuous dimension for the statistical analysis. With respect to cross-cultural comparison, it is important to note that measurement invariance has not been tested to confirm that the same construct is being measured across groups. Moreover, cultural dichotomy may have simplified the explanations attributed to differences and properties of the parental stress between the countries.

Given that couples were considered in this study, it would have been appropriate to include a measure of paternal involvement in childcare to include this variable as a covariate. Finally, we do not have a measure of child difficult behaviors and emotion regulation, which can strongly impact parenting stress and style. Future research should replicate our results using larger samples and including other Eastern and Western countries to investigate similarities and differences across cultures in families of children with ASD. We suggest that to examine individuals’ differences in stress and styles, the cultural dichotomic framework (individualism vs collectivism) should be extended by considering other crucial socio-cultural and individual domains. Since both mothers and fathers are included, dyadic statistical models can be used to account for the interdependence of the data. The assessment of parenting style can be improved by using standardized observational procedures and not only self-reported measures. This could help in identifying specific components of the parent–child relationship, including specific features, which characterize both maternal and paternal interactive styles. Moreover, longitudinal study designs are recommended to test predictive effects on parenting variables.

## Figures and Tables

**Table 1:** Characteristics of the participants.

	Italy	Japan	Group Differences
Child gender			p
Female	7 (14.9%)	9 (19.1%)	0.392
Male	40 (85.1%)	38 (80.9%)	0.392
Age child (months)	110.67 (23.72)	110.26 (20.54)	0.670
Autism severity			
Autism	24 (51.1%)	24 (51.1%)	0.582
Spectrum	23 (48.9%)	23 (48.9%)	0.582
Child IQ	89.49 (16.80)	91.60 (17.26)	0.563
Family SES	40.41 (12.61)	40.25 (9.64)	0.213
Age mothers (years)	42.17 (5.81)	41.11 (3.98)	0.469
Age fathers (years)	45.24 (5.73)	44.45 (6.53)	0.575

Note. IQ: intelligent quotient; SES: socioeconomic status measured with the Four-Factor Hollingshead Index.

**Table 2:** Descriptive statistics of variables of Italian and Japanese parents.

	Italy		Japan		F(1, 180)		
Variables	Mothers (*n* = 47)	Fathers (*n* = 45)	Mothers (*n* = 47)	Fathers (*n* = 42)	Country	Gender	Country × Gender
PSI-SF Parental Distress	24.11 (5.96)	24.69 (6.62)	31.17 (9.47)	28.66 (8.56)	22.60 ***	0.69	1.78
PSI-SF Parent–Child Dysfunctional Interaction	26.32 (6.53)	25.02 (5.95)	27.39 (6.52)	27.32 (7.11)	2.97	0.49	0.39
PSI-SF Difficult Child	32.43 (7.21)	29.93 (7.89)	35.57 (11.02)	33.66 (7.80)	7 **	2.87	0.05
PSI-SF Total Stress	82.85 (16.44)	79.64 (17.79)	94.13 (22.48)	89.63 (18.20)	14.17 ***	1.86	0.05
Parenting Social Style	4.15 (0.46)	3.79 (0.56)	3.83 (0.64)	3.58 (0.74)	8.67 **	12.05 ***	0.37

Note. * *p* < 0.05; ** *p* < 0.01; *** *p* ≤ 0.001; PSI-SF: Parenting Stress Index—Short Form.

**Table 3:** Hierarchical regression predicting total parenting stress.

	Total Parenting Stress					
	Model 1		Model 2		Model 3	
Variables	β	CI	β	CI	β	CI
Country	0.265 ***	4.73, 15.89	0.266 ***	4.72, 15.97	0.268 ***	4.91, 15.95
Parent gender			−0.110	−10.11, 1.56	−0.108	−9.90, 1.52
Parent Age			0.049	−0.36, 0.69	0.049	−0.35, 0.68
Family SES			−0.010	−0.25, 0.29	0.019	−0.23, 0.30
Child IQ					−0.039	−0.21, 0.12
Autism severity					0.213 **	2.72, 13.82
Δ*R*^2^	0.070 ***		0.012		0.049 *	
*R* ^2^	0.070		0.082		0.131	

Note. * *p* < 0.05; ** *p* < 0.01; *** *p* ≤ 0.001; SES family: socioeconomic status of the family; IQ: intelligent quotient.

**Table 4:** Hierarchical regression predicting social parenting style.

	Social Parenting Style							
	Model 1		Model 2		Model 3		Model 4	
Variables	β	CI	β	CI	β	CI	β	CI
Country	−0.200 **	−0.44, −0.07	−0.203 **	−0.44, −0.08	−0.209 **	−0.45, −0.08	−0.193 **	−0.41, −0.08
Parent gender			−0.253 ***	−0.51, −0.13	−0.252 ***	−0.51, −0.13	−0.280 ***	−0.52, −0.20
Parent Age			0.016	−0.02, 0.02	0.012	−0.02, 0.02	0.054	−0.01, 0.02
Family SES			−0.001	−0.01, 0.01	0.004	−0.01, 0.01	0.018	−0.01, 0.01
Child IQ					0.069	−0.003, 0.008	0.018	−0.004, 0.005
Autism severity					0.058	−0.11, 0.26	0.101	−0.04, 0.29
PSI-SF Parental Distress							0.131	0.002, 0.022
PSI-SF Parent–Child Dysfunctional Interaction							−0.578 ***	−0.07, −0.04
PSI-SF Difficult Child							0.048	−0.01, 0.02
Δ*R*^2^	0.040 **		0.062 **		0.007		0.248 ***	
*R* ^2^	0.040		0.102		0.109		0.356	

Note. * *p* < 0.05; ** *p* < 0.01; *** *p* ≤ 0.001; SES family: socioeconomic status; IQ: intelligent quotient; PSI-SF: Parenting Stress Index—Short Form.

## Data Availability

Not applicable.

## Supplementary Materials

The following are available online at https://www.mdpi.com/article/10.3390/brainsci11111419/s1, Table S1: Correlation between variables of Italian mothers. Table S2: Correlations between variables of Italian fathers. Table S3: Correlations between variables of Japanese mothers. Table S4: Correlations between variables of Japanese fathers.
