# Supplementary material for: Parenting Stress and Social Style in Mothers and Fathers of Children with Autism Spectrum Disorder: A Cross-Cultural Investigation in Italy and Japan
Source: Brain Sci. 2021 Oct 27;11(11):1419. doi: 10.3390/brainsci11111419 (PMC8615867; doi:10.3390/brainsci11111419)
Supplement: Supplementary file 1 [file brainsci-11-01419-s001.zip › brainsci-1414275-supplementary.pdf]

Table S1. Correlation between variables of Italian mothers

Variables	PSI-SF Parental Distress	PSI-SF Parent-Child Dysfunctional Interaction	PSI-SF Difficult Child	PSI-SF Total Stress	Parenting Social Style
PSI-SF Parental Distress	1	.575**	.436**	.782**	-.153
PSI-SF Parent-Child Dysfunctional Interaction	.575**	1	.617**	.876**	-,308*
PSI-SF Difficult Child	.436**	.617**	1	.841**	-.192
PSI-SF Total Stress	.782**	.876**	.841**	1	-.262
Parenting Social Style	-.153	-.308*	-.192	-.262	1

Note. PSI-SF: Parenting Stress Index – Short Form; * $p < 0.05$; ** $p < 0.01$.

Table S2. Correlations between variables of Italian fathers

Variables	PSI-SF Parental Distress	PSI-SF Parent-Child Dysfunctional Interaction	PSI-SF Difficult Child	PSI-SF Total Stress	Parenting Social Style
PSI-SF Parental Distress	1	.716**	.550**	.855**	-.145
PSI-SF Parent-Child Dysfunctional Interaction	.716**	1	.654**	.890**	-.348*
PSI-SF Difficult Child	.550**	.654**	1	.867**	-.133
PSI-SF Total Stress	.855**	.890**	.867**	1	-.229
Parenting Social Style	-.145	-.348*	-.133	-.229	1

Note. PSI-SF: Parenting Stress Index – Short Form; * $p < 0.05$; ** $p < 0.01$.

Table S3. Correlations between variables of Japanese mothers

Variables	PSI-SF Parental Distress	PSI-SF Parent-Child Dysfunctional Interaction	PSI-SF Difficult Child	PSI-SF Total Stress	Parenting Social Style
PSI-SF Parental Distress	1	.464**	.603**	.852**	-.144
PSI-SF Parent-Child Dysfunctional Interaction	.464**	1	.475**	.691**	-.588**
PSI-SF Difficult Child	.603**	.475**	1	.878**	-.075
PSI-SF Total Stress	.852**	.691**	.878**	1	-.271
Parenting Social Style	-.144	-.588**	-.075	-.271	1

Note. PSI-SF: Parenting Stress Index – Short Form; * $p < 0.05$; ** $p < 0.01$.

Table S4. Correlations between variables of Japanese fathers

Variables	PSI-SF Parental Distress	PSI-SF Parent-Child Dysfunctional Interaction	PSI-SF Difficult Child	PSI-SF Total Stress	Parenting Social Style
PSI-SF Parental Distress	1	.316*	.383*	.762**	-.055
PSI-SF Parent-Child Dysfunctional Interaction	.316*	1	.465**	.743**	-.668**
PSI-SF Difficult Child	.383*	.465**	1	.801**	-.402**
PSI-SF Total Stress	.762**	.743**	.801**	1	-.463**
Parenting Social Style	-.055	-.668**	-.402**	-.463**	1

Note. PSI-SF: Parenting Stress Index – Short Form; * $p < 0.05$; ** $p < 0.01$.
